# Epigenetic Mechanisms Shape the Biological Response to Trauma and Risk for PTSD: A Critical Review

**DOI:** 10.1155/2013/417010

**Published:** 2013-04-17

**Authors:** Morgan Heinzelmann, Jessica Gill

**Affiliations:** National Institute of Nursing Research, National Institutes of Health, Bethesda, MD 20814, USA

## Abstract

Posttraumatic stress disorder (PTSD) develops in approximately one-quarter of trauma-exposed individuals, leading us and others to question the mechanisms underlying this heterogeneous response to trauma. We suggest that the reasons for the heterogeneity relate to a complex interaction between genes and the environment, shaping each individual's recovery trajectory based on both historical and trauma-specific variables. Epigenetic modifications provide a unique opportunity to elucidate how preexisting risk factors may contribute to PTSD risk through changes in the methylation of DNA. Preexisting risks for PTSD, including depression, stress, and trauma, result in differential DNA methylation of endocrine genes, which may then result in a different biological responses to trauma and subsequently a greater risk for PTSD onset. Although these relationships are complex and currently inadequately described, we provide a critical review of recent studies to examine how differences in genetic and proteomic biomarkers shape an individual's vulnerability to PTSD development, thereby contributing to a heterogeneous response to trauma.

## 1. Introduction

Up to 90% of individuals experience a traumatic event at some time during their lives, yet most recover and suffer from no long-term ramifications; however, a subset of individuals develop posttraumatic stress disorder (PTSD) and are at high risk for health decline [1–4]. This heterogeneous response suggests that preexisting factors might influence how people respond to traumatic situations. We suggest that a more comprehensive examination of biomarkers that approximate PTSD risk would be of great value. Biomarker discovery in PTSD has been hindered by the lack of prospective studies in traumatized individuals, resulting in an insufficient understanding of the preexisting risk factors for PTSD onset as well as the mechanistic pathways that underlie this risk. Without this knowledge, we are unable to identify traumatized individuals at risk for PTSD development and, therefore, unable to implement effective, preventive interventions. Yet, additional biological analysis methods have become available, and these new strategies will be useful in addressing this critical issue. Specifically, the use of whole-genome gene expression and epigenetic modifications (DNA methylation) provide an opportunity to explore more than candidate biomarkers, to identify novel mechanisms related to PTSD risk, and to pinpoint genetic pathways that may be implicated in both risk and resilience to trauma.

A traumatic event places individuals at high risk for developing PTSD, resulting in rates of PTSD between 18% and 36% in trauma-exposed patients [1–4]; as mentioned above, most trauma-exposed individuals recover and do not develop PTSD. PTSD is viewed as a disorder of dysregulation of fear and processing of stimuli associated with trauma, and it is characterized by three main clusters of symptoms: reexperiencing, avoidance, and hyperarousal. Reexperiencing symptoms include distressing recollections or dreams of the event, flashbacks, and intense psychological and physiological reactivity to internal and external cues; avoidance symptoms include evasion of any thoughts or feelings of people and places that remind the individual of the traumatic event, decreased interest in participating in activities, and emotional numbing; and hyperarousal symptoms include problems falling or staying asleep, irritability or anger, hypervigilance, and exaggerated startle reflex. It has become increasingly clear that environmental influences early in development remain pervasive into adulthood, a relationship that is attributed to an interaction of gene function and environment. Both genetic and environmental factors are critical to developmental processes, and even minor changes in either type of factor can result in trajectories of resilience or vulnerability [5]; however, it is the interaction between these factors that may provide the most vital information to understanding the heterogeneous response to trauma. Epigenetic modifications occur in response to an environmental factor and include DNA methylation, acetylation, and histone modification which alter DNA accessibility and chromatin structure, thereby regulating activity of the gene in a long-lasting manner. Because the risk for PTSD onset is influenced by environmental factors that predate trauma exposure, epigenetics may provide novel insights into the heterogeneous response to a trauma. Large epidemiological studies link pretrauma risks including previous depression, stressors, and traumatic events to a greater risk for PTSD onset following trauma exposure [6], suggesting that these factors may contribute to variability of individuals in response to a trauma.

The support for the mediating link of epigenetic modifications and PTSD vulnerability is reported in preclinical studies. Specifically, preclinical models illustrate these complex relationships and link epigenetic modifications in neurons to psychological vulnerability following stressors. In rats, the offspring of the high caring mothers (i.e., high licking) exhibit the reduced methylation of the glucocorticoid receptor gene [7] and endocrine regulation of a subsequent stressor [8]. In contrast, in non-human primates, offspring that face early adversity exhibits endocrine dysregulation [9] as well as reductions in neuronal plasticity in the prefrontal cortex that persist into adulthood [10]. Early stressors in humans have also been linked to epigenetic modifications, including increased methylation of the glucocorticoid promoter in hippocampal neurons of suicide completers with histories of early childhood maltreatment [11]. Cord blood collected from mothers with high levels of depression and anxiety during the third trimester displays similar glucocorticoid methylation differences, which are linked to a dysregulation of the endocrine system [12]. In studies of rats who exhibit PTSD-like behavior, there is evidence of increased methylation of stress response genes including brain-derived neurotrophic factor [13] and nuclear protein phosphate-1 [14] in neurons, which result in the onset of PTSD-like behavior [15]. These preclinical studies provide insights into the heterogeneous response to trauma and stress and suggest that epigenetic modifications in neurons result in the onset of PTSD [16, 17].

**Figure 1:**
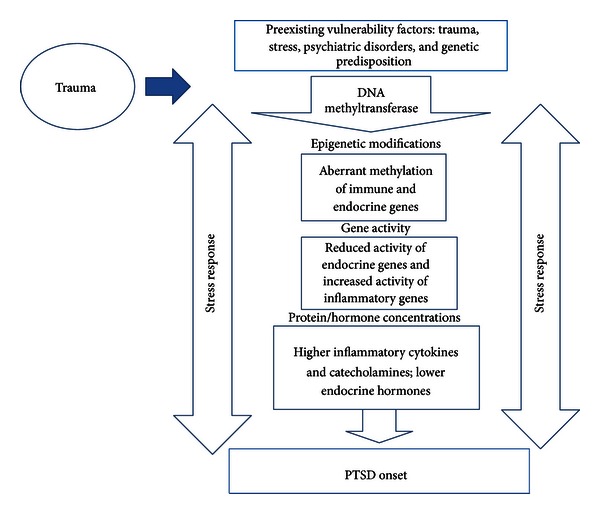
Epigenetic mechanisms of PTSD onset.

We suggest that the heterogeneous response to trauma relates to complex interactions between genes and the environment, shaping each individual's recovery trajectory based on both historical and trauma specific variables (see Figure 1). One of the major environmental factors linked to epigenetic changes is stress, which is a critical factor in the pathogenesis of PTSD. There is robust evidence linking PTSD onset to epigenetic changes following stress; yet this evidence is only starting to accumulate from the clinical studies. Recent advances in laboratory analyses and biostatistical methods provide new opportunities to determine the mechanisms of PTSD onset; however, many of these advances are not yet applied. Additionally, current studies are limited by our inability to determine pretrauma epigenetic status as well as our restriction to peripheral blood samples in clinical samples. Here, we provide a critical review of recent studies to examine how differences in genetic and proteomic biomarkers may underlie the vulnerability of the individual to develop PTSD, and how this field of study may provide fundamental insights into the identification of individuals at risk for PTSD development following trauma and the development of novel, interventional strategies.

## 2. Methods

In order to obtain a comprehensive pool of prospective studies investigating peripheral biomarkers of PTSD following trauma, PubMed, OVID/MEDLINE, the Cochran Database, Embase, Scopus, CINAHL, and PsychInfo were systematically searched using the following National Library of Medicine Medical Subject Headings (MeSH) “posttraumatic stress disorder,” “genes,” “cytokines,” “neuropeptides,” and “inflammation.” Additionally, each key word was cross-referenced with each other in each of the various databases. The search extended to the literature published between 2000 and 2013, and studies were included in the review if they (1) were primary research articles, (2) were published in English, (3) were conducted in humans, and (4) examined epigenetic modifications (i.e., DNA methylation), gene expression, or a proteomic biomarker that was linked to PTSD risk following a trauma. The following components and variables of interest were appraised to provide an overall synthesis of the available literature: study purpose, design, methods, sample size, demographics, type of trauma, severity of injuries sustained (mild, moderate, and severe), type of biological sample collected, and times of collection following trauma. We were able to locate 6 clinical studies that examined epigenetic modifications (i.e., DNA methylation), 9 studies of gene expression, and 14 studies that used a proteomic biomarker to examine the risk for PTSD onset (3 pediatric and 9 adult studies). All studies were reviewed and are presented in Tables 1, 2, and 3.

### 2.1. Epigenetics

Clinical studies are restricted to examining epigenetic modifications in samples of peripheral blood, but these studies do provide some key insights into how these molecular changes relate to PTSD risk (see Table 1). Specifically, these studies implicate insufficient regulation of inflammatory activity and reduced neurotransmitter activity in PTSD risk. In support of this, studies in chronic PTSD-affected patients report hypermethylation of inflammatory initiator genes (toll-like receptors 1 & 3, IL-8, chemokine ligand 1, and others) and demethylation of inflammatory regulatory genes (FK506 binding protein-5 [FKBP5]) [18]. In a subsequent study, Ressler et al. (2011) linked these molecular-genetic mechanisms to higher concentrations of inflammatory cytokines in patients with chronic PTSD [19]. Together, these association studies suggest that observations by us and others of excessive inflammation in chronic PTSD [20, 21] likely relate to these DNA methylation differences; however, their cross- sectional designs prohibit linking these molecular differences to PTSD vulnerability.

In contrast, a recent study measured DNA methylation and reported that postdeployment hypomethylation of LINE-1 was associated with PTSD onset following deployment [25]. The authors of this report postulate that LINE-1, a regulator of stress response in the immune system, may represent an adaptive response to combat stress that protects against PTSD; yet additional studies are needed in more generalized samples that include women and various traumatic events. In addition, this study did not determine predeployment factors such as previous PTSD, depression, or trauma that contributed to precombat methylation differences, resulting in an inability to understand how the interactions between genes and environment prior to trauma underlies PTSD risk.

Other studies suggest that differential methylation of neurotransmitter genes is linked to PTSD onset. In two studies using samples of civilians from the Detroit Neighborhood Health Study, neurotransmitter genes were implicated in the risk for PTSD development. In one study that examined the serotonin transporter gene (SLC6A4), methylation levels were modified by the effect of the number of traumatic events on PTSD after controlling for SLC6A4 genotype, such that persons with more traumatic events were at increased risk for PTSD, but only at lower methylation levels. At higher methylation levels, individuals who reported more traumatic events were protected from this disorder, suggesting that the serotonin transporter gene may also be important in trauma-related resilience [22]. In a study using the same patient sample, the candidate gene MAN2C1 showed a significant methylation × trauma experience interaction such that those with both higher MAN2C1 methylation and greater exposure to traumatic events showed an increase in risk of lifetime PTSD [24]. Although these studies provide unique insights into risks for PTSD based on DNA methylation, trauma, and psychiatric-related symptoms, these studies used cross-sectional design and did not include definitive diagnoses determined through a clinical interview.

Although not epigenetic in nature, previous studies that examine genetic inheritance provide further evidence of genetic underpinnings in endocrine modulating genes in the risk for PTSD. In brief, previous studies link single nucleotide polymorphisms (SNPs) of FKBP5, a negative regulator of GCR sensitivity to a greater risk for PTSD onset [29, 49, 50]. Current findings indicate that genetic predisposition differences in another key inflammatory gene, corticotrophin- releasing hormone type 1 receptor gene (CRHR1), increase PTSD onset in children who were abused at an early age [51]. With pediatric injury patients, a longitudinal study identified one SNP significantly related to acute PTSD symptoms as well as trajectory of symptoms over time [52]. These findings are meaningful in terms of genetic and environmental interplay producing risk for PTSD; however, current studies do not explicate the link between baseline epigenetics in immune regulatory genes and the risk for PTSD onset following a trauma. Yet, taking these studies together with studies that examine gene function, there is evidence that a combination of genetic and environmental factors contributes to psychological vulnerability following a trauma by shaping an individual's neuronal and biological stress response, warranting future prospective studies to determine these important temporal relationships [53].

### 2.2. Gene Expression

Nine studies have also used gene expression analysis of peripheral blood to examine the mechanisms of PTSD, providing additional insights into the complex biological processes underlying PTSD onset. Gene expression involves processes that alter the ultimate product of the gene, which is most often the production of proteins. Current studies using gene expression analysis methods also support the role of insufficient inflammatory regulation in the risk for PTSD onset, by implicating higher expression of inflammatory genes and lower expression of genes that regulate inflammatory processes (see Table 2). In a recent cross-sectional study of PTSD patients, a reduction in the transcriptional activity of genes that regulate inflammation including FKBP5 and IL18 and STAT pathways was reported in an urban sample of primarily African American participants [30]. Reductions in gene expression of STAT5B and MHC II class receptor genes in participants who developed PTSD following the 9-11 terrorist attacks were also reported [29]. Also, in a sample of participants who developed PTSD following 9-11, reduced FKBP5 gene expression was most related to PTSD severity in regression analysis as well as a reduction in STAT5B, a direct inhibitor of GR, and a major histocompatibility complex (MHC) class II gene expression; however, significant differences were related to PTSD symptom severity and not a definitive diagnosis of PTSD [27]. Lastly, in a recent study of 12 women with PTSD related to child abuse, increased glucocorticoid receptor sensitivity in monocytes was linked to increased NF-*κ*B activity; however, this study did not use a whole-genome analysis like the other reviewed studies, but instead a DNA binding ELISA in mononuclear cells [31]. Thus, together, these studies suggest that reduced gene expression of immune regulatory genes is associated with PTSD but is limited to small sample sizes and cross-sectional design.

In contrast, two recent studies provide vital insights into how gene expression differences contribute to PTSD onset through the use of a prospective design in male service members who are evaluated prior to and then following deployment. In a study of 448 male soldiers, predeployment high glucocorticoid receptor numbers and low FKBP5 mRNA expression were associated with increased risk for a high level of PTSD symptoms [32]. In a similar sample, a high level of PTSD symptoms after deployment was independently associated with a high DEX sensitivity of T-cell proliferation before deployment, but only in individuals who reported PTSD symptoms without depressive symptoms [33]. These studies link alterations in gene expression to functional immune cell changes. Importantly, these studies also link preexisting biological differences in cell function and gene expression to the risk for PTSD onset following a trauma. These studies are limited by their use of gene expression analysis of only predetermined target genes, restricting our ability to identify novel biological targets for PTSD risk and to understand how known risks relate to less well known genes related to PTSD risk. Although these studies have some limitations, they provide fundamental information regarding the mechanisms of PTSD onset. Specifically, these studies report that it is likely an accumulation of preexisting factors that result in differential gene activity and vulnerability to develop PTSD following trauma exposure.

### 2.3. Proteomics

Studies of proteomic biomarkers of PTSD risk have been undertaken for far longer than studies that use genetic analyses; yet they have been limited by many methodological issues. These issues include an inability to control for timing of the sample collection, with most proteins exhibiting substantial circadian variation, the impact of the physical consequences of trauma, and differences in the timing between the occurrence of the trauma and the biological sample. Despite these limitations, these studies provide some vital insights into the biological mechanisms underlying PTSD vulnerability. Proteins play important roles in both intracellular processes and intercellular communication, thereby contributing to the orchestration of responses to traumatic events. Thus, proteins should be considered as possible PTSD biomarkers. Studies of acutely traumatized individuals suggest that high concentrations of inflammatory proteins are linked to PTSD onset. Here, we review 14 studies that collect a biological sample within the acute period following a trauma and use this biomarker to approximate PTSD risk (see Table 3).

In children following a motor vehicle accident (MVA), high concentrations of IL-6 twenty four hours after the MVA predicted PTSD onset at six months [37]; however, these elevations were no longer evident in those children who developed PTSD. Similar findings are reported in a study of adults hospitalized for orthopedic injuries, reporting that high concentrations of IL-6 as well as IL-8 related to the risk acute stress disorders symptoms; however, this study did not link these biomarkers to the risk for PTSD onset [47]. IL-8 is a mediator of the innate immune system, with higher concentrations indicating a greater immune challenge. In contrast, low concentrations of IL-8 predicted high PTSD symptoms following the earthquakes in China, whereas high IL-6 predicted anxiety and depression symptoms but was not specific to PTSD [54]. The Song et al. [54] study differs from the other two studies as biomarker collection was within months, not hours of the trauma, and many of the participants did not sustain physical injuries. A benefit of the Song study was the evaluation of cooccurring depression with PTSD, which is a limitation of the other prospective studies. In studies of chronic PTSD, cooccurring depression is associated with higher concentrations of IL-6 [20, 55]. Therefore, inflammation in the acute recovery period may be very detrimental to psychological recovery as high concentrations of inflammatory cytokines exert central effects including the induction of “sickness behavior” [56–60]. In addition, inflammatory cytokines increase blood brain barrier disruption [61–63] and alter metabolism of serotonin [64–67], all of which contributes to neuronal vulnerability despite of the excessive central inflammatory actions, including overactivation of microglia [68–70]. Together, these studies suggest that increased concentrations of inflammatory cytokines relate to the risk for PTSD onset.

There is also evidence that endocrine dysregulation relates to PTSD risk; however, this risk may relate to previous trauma. In three studies of children recruited from emergency departments, high cortisol measured in the urine predicted acute stress symptoms at six weeks following the trauma; however, in the Ostrowski et al. (2007) study high cortisol predicted PTSD onset in boys only [36]. This is of interest as studies of adult women report that it is low cortisol concentrations that predict PTSD onset [39]. Resnick et al. (1995) support this finding in a similar sample but reports that this relationship only relates to those women who report a trauma prior to the rape [38]. In samples of men and women following an MVA, low cortisol concentrations are also linked to PTSD risk [40, 44]; however, the role of previous trauma is not reported in these studies. Other studies do not link cortisol concentrations to PTSD risk [39, 41, 42, 45, 46]. In a unique study of service members prior to and following deployment, concentrations of cortisol were not related to PTSD risk; however, greater glucocorticoid binding in mononuclear cells prior to deployment predicted PTSD onset, suggesting that systems that regulate cortisol activity relate to PTSD risk [48].

ANS dysregulation contributes to PTSD vulnerability [71, 72]; however, current biomarker studies that measure catecholamines or metabolites have not supported this relationship. Studies that examined catecholamine concentrations from blood samples did not significantly differ in studies of adults [39, 46] or children [35, 37]. Studies of concentrations of catecholamines using urine samples also did not show significant findings in adults [35, 40, 42, 46] or in children [35]. Therefore, ANS dysregulation may relate to PTSD risk yet this is not supported in biomarkers studies; this discrepancy may be related to the high degree of turnover of catecholamines in circulation, in which current studies may not be able to capture. Future studies that are able to approximate ANS dysregulation will be important as catecholamines influence immune and endocrine function, and also neuronal circuitry required to psychologically cope with trauma [73].

## 3. Conclusions

In this review, we provide a comprehensive examination of the biomarkers that contribute to PTSD risk. We suggest that epigenetic modifications shape the resulting proteomic response of the individual through differential gene activity, thereby contributing to the heterogeneous response to trauma and differential risk for PTSD onset. Additional studies are needed to elucidate these relationships and to design technological methods to use these biomarkers to identify trauma patients at the highest risk for PTSD onset. Preventative interventions would be of great value in patients at the highest risk for PTSD to prevent chronic symptomatology. In addition, these studies may inform the development of novel interventions including pharmacological agents that are better able to prevent the onset of PTSD.

Addressing this issue is critical, as delivering effective, preventive interventions for PTSD could save the USA up to 180 million dollars each year in health care costs [74–79] and the lives of up to 9,000 individuals from suicide [80–83]. Health care providers have an opportunity to reduce the risk for PTSD onset as up to 3 million adults seek immediate medical care for traumatic injuries each year [84], resulting in the onset of PTSD in almost 1 million of Americans annually [1, 4, 85–87]. However, even in this group, there is a high level of interindividual response variability to traumatic injuries, suggesting that a biomarker that is able to approximate PTSD risk would be of great value in directing preventive measures. Preventing PTSD is paramount and would reduce the substantial morbidity and health-related mortality associated with this devastating disorder [2, 85].

## Figures and Tables

**Table 1:** Epigenetics studies.

Study	Analysis method	Sample	Findings
Uddin et al., 2010 [18]	DNA methylation; >14,000 genes	100 individuals from DNHS (PTSD = 23)	PTSD had greater methylation of toll-like receptors 1 and 3, IL-8, and others compared to controls and had a greater overall number of uniquely methylated genes.

Koenen et al., 2011 [22]	DNA methylation and genotype; *SLC6A4 *	100 individuals from DNHS (PTSD = 23)	Neither genotype nor methylation of *SLC6A4* was associated with PTSD; however, when controlling with genotype, lower methylation levels were associated with increased risk for developing PTSD among individuals with more traumatic events.

Ressler et al., 2011 [19]	DNA methylation; 44 SNPs of *PACAP* and *PAC1 *	64 individuals, primarily African American (PTSD = 24)	An SNP in *ADCYAP1R1*, rs2267735, predicted PTSD diagnosis and symptoms in women; methylation of this gene was also associated with PTSD.

Smith et al., 2011 [23]	DNA methylation; global and site specific	110 African Americans (PTSD = 50)	Global methylation was increased in subjects with PTSD, as compared to control subjects or subjects with a history of childhood trauma; CpG sites in *TPR*, *CLEC9A*, *APC5*, *ANXA2*, and *TLR8* were differentially methylated in subjects with PTSD.

Uddin et al., 2011 [24]	DNA methylation; 33 candidate genes	100 individuals from DNHS (PTSD = 23)	One candidate gene, *MAN2C1*, showed significantly higher methylation in subjects with lifetime PTSD.

Rusiecki et al., 2012 [25]	DNA methylation; LINE-1 and Alu	150 service members (PTSD = 75)	LINE-1 was hypomethylated in PTSD versus controls postdeployment and hypermethylated postdeployment versus predeployment in controls; Alu was hypermethylated in PTSD versus controls predeployment.

**Table 2:** Gene expression studies.

Study	Analysis method	Sample	Findings
Zieker et al., 2007 [26]	Pre-selected stress-immune genes, whole-blood	16 individuals (PTSD = 8)	In PTSD, upregulated (4): glutamate transported, IGF-2; downregulated (14): IL-18, IL-16, colony stimulating factor.

Yehuda et al., 2009 [27]	Whole blood gene expression	35 individuals exposed to 9/11 (PTSD = 15)	FKBP5, STAT5B, and MHC class II showed reduced expression in individuals with PTSD.

Neylan et al., 2011 [28]	CD14+ monocyte; gene expression	67 ± trauma-exposed individuals (PTSD = 34)	In PTSD patients, three monocyte genes were underexpressed in men but not in women.

Sarapas et al., 2011 [29]	Genome-wide gene expression	40 individuals exposed to 9/11 (PTSD = 20)	PTSD patients showed a reduction in gene expression of STAT5B and nuclear factor I/A.

Mehta et al. 2011 [30]	Whole-blood gene expression and SNP of FKBP5	211 low income (PTSD = 75)	With FKBP5 SNP added to interaction with PTSD, there was a reduction in 32 genes including IL-18 and STAT pathway.

Pace et al., 2012 [31]	Nuclear factor-*κ*B activity in peripheral blood	36 women (PTSD = 12)	Increased nuclear factor-*κ*B activity was associated with women with PTSD as compared to controls.

van Zuiden et al., 2012 [32]	GR number; FKBP5, GILZ, and SGK1 mRNA expression	448 military personnel (PTSD = 35)	Predeployment high GR number, low FKBP5 mRNA expression, and high GILZ expression predicted PTSD development.

van Zuiden et al., 2012 [33]	GC sensitivity of leukocytes	526 military personnel (PTSD = 46)	Predeployment sensitivity of GCRs on leukocytes predicted development of PTSD.

Matić et al., 2013 [34]	GR function and expression using PCR	347 ± war trauma-exposed individuals (PTSD = 113)	Lower GR sensitivity in PBMCs and low gene-expression of GR were found in PTSD patients.

**Table 3:** Proteomic studies.

Pediatric studies	Sample	Collection; followup	Outcome	Results
Delahanty et al., 2005 [35]	58 ED patients	ED; 6 wks	Diagnosis	High cortisol and epinephrine predicted acute PTSD symptoms.
Ostrowski et al., 2007 [36]	54 ED patients	ED; 6 wks, 7 mos	Diagnosis	High cortisol predicted acute PTSD symptoms and PTSD onset in boys.
Pervanidou et al., 2007 [37]	56 MVA patients (9 = PTSD)	ED; 1, 6 mos	Diagnosis	High cortisol and IL-6 predicted PTSD onset.

Adult studies	Sample	Collection; followup	Outcome	Results

Resnick et al., 1995 [38]	37 rape survivors (19 = PTSD)	ED; 17–157 days	Diagnosis	Low cortisol in previously assaulted women predicted PTSD onset.
Yehuda et al., 1998 [39]	20 rape survivors (11 = PTSD)	ED; 27–157 days	Diagnosis	Cortisol and MHPG did not predict PTSD onset.
Delahanty et al., 2000 [40]	99 MVA patients (9 = ASD)	ED; 1 mo	Diagnosis	Low cortisol predicted acute PTSD symptoms.
Bonne et al., 2003 [41]	21 ED patients (8 = PTSD)	1 wk; 6 mos	Diagnosis	Cortisol did not predict PTSD onset.
Heinrichs et al., 2005 [42]	43 firefighters (7 = PTSD at 2 yrs)	Training; 6, 9, 12, 24 mos	Symptom report	Cortisol and CA did not predict PTSD onset.
McFarlane et al., 1997 [43]	40 MVA patients (7 = PTSD)	ED; 2, 10 days and 6 mos	Diagnosis	Low cortisol predicted PTSD onset.
Ehring et al., 2008 [44]	53 MVA patients (5 = PTSD)	ED; 2 wks, 6 mos	Diagnosis	Low cortisol predicted PTSD onset.
Shalev et al., 2008 [45]; Videlock et al., 2008 [46]	155 ED patients (31 = PTSD)	ED; 10 days, and 1, 5 mos	Diagnosis	Cortisol, ACTH, GR, and NE did not predict PTSD onset.
Cohen et al., 2011 [47]	48 orthopedic patients, 13 HC	At hospitalization; 1 mo	Symptom report	High IL-8 and low TGF-*β* predicted acute PTSD symptoms.
van Zuiden et al., 2011 [48]	68 service members, (34 = PTSD)	Prior; following deployment	Symptom report	High GR predicted PTSD onset.
